# Unraveling the Challenges: A Critical Review of Congenital Malformations in Low Socioeconomic Strata of Developing Countries

**DOI:** 10.7759/cureus.41800

**Published:** 2023-07-12

**Authors:** Nidhi Shetty, Saket Mantri, Sristy Agarwal, Ashwini Potdukhe, Mayur B Wanjari, Avinash B Taksande, Seema Yelne

**Affiliations:** 1 Community Medicine, Jawaharlal Nehru Medical College, Datta Meghe Institute of Higher Education and Research, Wardha, IND; 2 Medicine, Jawaharlal Nehru Medical College, Datta Meghe Institute of Higher Education and Research, Wardha, IND; 3 Medical Surgical Nursing, Srimati Radhikabai Meghe Memorial College of Nursing, Datta Meghe Institute of Higher Education and Research, Wardha, IND; 4 Research and Development, Jawaharlal Nehru Medical College, Datta Meghe Institute of Higher Education and Research, Wardha, IND; 5 Physiology, Jawaharlal Nehru Medical College, Datta Meghe Institute of Higher Education and Research, Wardha, IND; 6 Nursing, Shalinitai Meghe College of Nursing, Datta Meghe Institute of Higher Education and Research, Wardha, IND

**Keywords:** equity, healthcare access, factors influencing, prevalence, developing countries, low socio-economic strata, congenital malformations

## Abstract

Congenital malformations pose significant challenges in the low socioeconomic strata of developing countries. This review critically examines the prevalence, patterns, and factors influencing congenital malformations in these settings. It explores the physical, psychological, and economic consequences for affected individuals and their families and the social stigma and discrimination they face. The review highlights the importance of equity and access to healthcare services, the role of environmental factors and nutritional deficiencies, and the ethical considerations and policy implications associated with congenital malformations. Existing interventions, challenges in implementation, and innovative approaches are discussed. Gaps in knowledge and areas for further research are identified. Addressing congenital malformations in low socioeconomic strata requires multidisciplinary collaboration, advocacy, and inclusive policies. By prioritizing preventive measures, early detection, and comprehensive care, the burden of congenital malformations can be reduced, improving the quality of life for affected individuals and their communities.

## Introduction and background

Congenital malformations, or congenital disabilities, are structural or functional abnormalities during fetal development. They can profoundly impact the health and well-being of individuals, families, and communities. While congenital malformations affect populations worldwide, the burden is disproportionately higher in developing countries, particularly among low socioeconomic strata [1,2]. Developing countries face numerous challenges in addressing congenital malformations, including limited access to healthcare services, inadequate prenatal care, and environmental factors contributing to congenital disabilities. These challenges are further exacerbated by the social and economic disparities in low socioeconomic strata, where individuals and families often struggle with poverty, limited education, and lack of resources [3-5].

Congenital malformations are a significant public health concern in developing countries, where they contribute to a substantial burden of disease and disability. These countries often lack the resources and infrastructure to provide adequate healthcare services and interventions for affected individuals. As a result, congenital malformations can have long-lasting physical, psychological, and socioeconomic consequences [6]. The types and prevalence of congenital malformations vary across developing countries and regions, influenced by genetic factors, environmental exposures, and social determinants. Common congenital disabilities include neural tube defects, congenital heart diseases, cleft lip and palate, and limb abnormalities. Understanding the specific patterns and distribution of congenital malformations in developing countries is essential for effective prevention and management strategies [7,8].

Low socioeconomic strata within developing countries represent a vulnerable population that experiences significant disparities in access to healthcare, education, and socioeconomic opportunities. These individuals often face multiple challenges, such as inadequate nutrition, limited prenatal care, exposure to environmental toxins, and a lack of awareness about preventive measures [9]. By focusing on the low socioeconomic strata, this review aims to highlight the challenges faced by individuals and communities with limited resources. Understanding the social, economic, and environmental factors unique to this population is crucial for developing targeted interventions and policies to address congenital malformations' underlying causes and improve health outcomes [10].

This review aims to critically examine the challenges associated with congenital malformations in the low socioeconomic strata of developing countries. By synthesizing existing literature and research, this review aims to shed light on the unique issues individuals and communities face in these settings and provide a comprehensive understanding of the factors contributing to the high prevalence of congenital malformations.

## Review

Prevalence and patterns of congenital malformations

Global Prevalence Rates of Congenital Malformations

Congenital malformations are a significant global health concern affecting populations worldwide. These malformations encompass various structural and functional abnormalities present at birth. Estimating the global prevalence of congenital malformations is complex due to variations in data collection, reporting systems, and diagnostic capabilities among countries [11]. Studies have reported varying prevalence rates, but general estimates suggest that approximately 3-6% of newborns are born with congenital malformations. However, it is important to note that prevalence rates can vary significantly based on various factors. Geographical location plays a role, with some regions experiencing higher rates than others. Genetics, environmental exposures, and maternal health can contribute to regional variations in prevalence [12].

Socioeconomic factors also influence the prevalence rates of congenital malformations. In developing countries, individuals from low socioeconomic strata typically experience higher congenital malformations than their counterparts in higher socioeconomic or developed nations [12]. Limited access to healthcare services, inadequate prenatal care, poor nutrition, and exposure to environmental hazards contribute to the increased occurrence of congenital malformations in these populations [13]. Access to healthcare plays a crucial role in determining the prevalence of congenital malformations. Developing countries with limited healthcare infrastructure and resources often face challenges in providing comprehensive prenatal care, genetic counseling, and specialized interventions for individuals with congenital malformations. This lack of access can lead to underreporting or underdiagnosis of cases, potentially underestimating the true prevalence rates [14].

Focus on Developing Countries and Low Socioeconomic Strata

Developing countries, particularly those within low socioeconomic strata, are confronted with a significant burden of congenital malformations. These countries often lack the resources, infrastructure, and healthcare systems to effectively address and manage these conditions. Congenital malformations are higher in these settings due to various factors [15]. Limited access to prenatal care is a major contributing factor [16]. In low socioeconomic strata, pregnant women often face barriers to accessing quality healthcare services, including inadequate antenatal care and limited availability of skilled healthcare professionals. Late or no prenatal screenings and insufficient follow-up care further hinder the early detection and management of congenital malformations [16].

Inadequate nutrition also plays a role. Nutritional deficiencies and poor diet choices are seen in both developed and developing countries during pregnancy. Insufficient intake of vital nutrients, including folic acid, iron, and iodine, may heighten the risk of congenital disabilities and hinder fetal development [17]. Exposure to environmental hazards is another contributing factor. Developing countries may have higher levels of pollution, poor sanitation systems, and exposure to hazardous substances due to industrial activities and limited environmental regulations. These ecological risks can harm fetal development, increasing the likelihood of congenital malformations [18].

Socioeconomic disparities further exacerbate the problem. Poverty, limited education, inadequate housing, and lack of clean water and sanitation are prevalent in low socioeconomic strata. These socioeconomic determinants impact maternal health, increase the risk of adverse pregnancy outcomes, and contribute to the higher prevalence rates of congenital malformations [19]. Addressing the challenges faced by developing countries and low socioeconomic strata is crucial to reducing the congenital malformations burden. Efforts should focus on improving healthcare infrastructure, increasing access to prenatal care, promoting proper nutrition during pregnancy, and implementing measures to reduce exposure to environmental risks. Addressing socioeconomic disparities through poverty alleviation, education, and social support programs is essential for preventing and managing congenital malformations in these vulnerable populations [20].

Common Types of Congenital Malformations in Developing Countries

Certain congenital malformations are more prevalent in developing countries, reflecting a combination of genetic, environmental, and socioeconomic factors. Understanding the common congenital malformations in these settings is crucial for effective prevention, detection, and management [21].

Neural tube defects: Prevalence of neural tube defects in more than 300,000 babies who are born with a neural tube defect each year [22]. Neural tube defects, such as spina bifida and anencephaly, are among developing countries' most frequently reported congenital malformations. These occur due to incomplete neural tube closure during early fetal development. Factors such as insufficient intake of folic acid, nutritional deficiencies, and exposure to certain environmental factors contribute to the higher prevalence of neural tube defects in low socioeconomic strata [22].

Congenital heart defects: The birth prevalence of CHD is reported to be 8-12/1000 live births [23]. Congenital heart defects are another common malformation observed in developing countries. These structural abnormalities of the heart can range from mild to severe, impacting the heart's structure and function. Genetic factors, maternal health conditions, and exposure to certain infections during pregnancy can contribute to developing congenital heart defects. Limited access to specialized cardiac care and interventions in developing countries can pose additional challenges for affected individuals [23].

Cleft lip and palate: The annual prevalence of infants born with cleft lip with or without cleft palate is 10 in 10,000 [24]. Cleft lip and palate are facial malformations characterized by incomplete fusion of the mouth's lip and roof during fetal development. These conditions can cause feeding, speech, and oral health difficulties. Genetic and environmental factors, including maternal smoking and nutritional deficiencies, contribute to cleft lip and palate occurrence. Timely surgical interventions and multidisciplinary care are essential for managing these malformations [24].

Limb abnormalities: The prevalence rates of congenital limb defect (CLD) vary widely among countries, from 1-4 per 1000 live births [25]. Limb abnormalities, such as missing or underdeveloped limbs (amelia and phocomelia) or malformations of the hands or feet (such as polydactyly or clubfoot), are commonly observed in developing countries. Genetic factors, exposure to certain medications or toxins during pregnancy, and limited access to appropriate prenatal care contribute to the higher prevalence of limb abnormalities in low socioeconomic strata [25].

Gastrointestinal malformations: Gastrointestinal malformations incidence was 25.2 cases a year, such as esophageal atresia, intestinal atresia, and anorectal malformations, are significant congenital abnormalities affecting the digestive system. These malformations can cause feeding difficulties and digestive problems and may require surgical interventions. Environmental factors, genetic predisposition, and maternal health conditions can contribute to the development of gastrointestinal malformations [26]. Identifying and addressing the common congenital malformations in developing countries is essential for implementing targeted interventions, improving access to specialized care, and supporting affected individuals and their families. Prevention strategies, early detection through prenatal screenings, and multidisciplinary care approaches can help mitigate the impact of these malformations and improve the overall well-being of affected individuals [27].

Regional or Cultural Variations in Patterns

Patterns of congenital malformations can exhibit regional or cultural variations within developing countries. Genetic predisposition, consanguinity, exposure to specific teratogens, and cultural practices may contribute to these variations [28]. Understanding the prevalence and patterns of congenital malformations is crucial for effective prevention, management, and resource allocation in developing countries, particularly within low socioeconomic strata. By recognizing the specific types and regional variations in these settings, healthcare systems and policymakers can develop targeted interventions to address these populations' unique challenges associated with congenital malformations [29].

Factors influencing congenital malformations in low socioeconomic strata

Socioeconomic Determinants of Congenital Malformations

Socioeconomic determinants of congenital malformations refer to the underlying social and economic factors contributing to congenital disabilities in low socioeconomic strata. These factors significantly impact the health and well-being of individuals and communities [30]. Poverty is one of the primary socioeconomic determinants associated with higher rates of congenital malformations. Limited financial resources can lead to inadequate nutrition, lack of access to healthcare services, and substandard living conditions, increasing the risk of congenital disabilities. Poor maternal nutrition during pregnancy, resulting from limited access to nutritious food, can negatively affect fetal development and contribute to the occurrence of malformations [31].

Limited education is another socioeconomic determinant that plays a role in congenital malformations. In low socioeconomic strata, individuals may have restricted access to education, leading to lower health literacy and awareness about preventive measures. This can result in a lack of knowledge regarding the importance of prenatal care, proper nutrition, and avoiding harmful substances during pregnancy, increasing the risk of congenital disabilities [32]. Inadequate housing and lack of access to clean water and sanitation are additional socioeconomic determinants contributing to congenital malformations. Living in substandard housing increases the risk of exposure to environmental hazards, such as indoor air pollution, mold, and pests, which can adversely affect fetal development. Lack of access to clean water and sanitation facilities can also increase the risk of infectious diseases during pregnancy, which may result in congenital disabilities [33].

Furthermore, these socioeconomic determinants can impact maternal health, as women in low socioeconomic strata may have limited access to healthcare services and face barriers in seeking timely and appropriate prenatal care. Delayed or inadequate prenatal care can hinder the early detection and management of congenital disabilities, potentially leading to more severe health outcomes for both the mother and the child [34]. Addressing socioeconomic determinants of congenital malformations requires a comprehensive approach that includes poverty reduction strategies, improving educational opportunities, enhancing housing conditions, and ensuring access to clean water and sanitation facilities. Efforts should also focus on improving healthcare access and promoting awareness about the importance of proper nutrition and prenatal care in low socioeconomic strata. Addressing these socioeconomic determinants can mitigate the risk of congenital malformations, leading to improved maternal and child health outcomes [35].

Access to Healthcare and Prenatal Services in Developing Countries

Access to healthcare and prenatal services in developing countries is a crucial aspect that significantly affects the occurrence and management of congenital malformations in low socioeconomic strata. In these settings, individuals often face limited access to healthcare facilities and services, resulting in inadequate antenatal care and reduced early detection and intervention opportunities [36]. Inadequate antenatal care is a common issue, with pregnant women in low socioeconomic strata having limited opportunities for regular check-ups, screenings, and consultations with healthcare professionals. This lack of comprehensive prenatal care can delay identifying potential congenital disabilities or abnormalities in fetal development, making it challenging to implement timely interventions [37].

Late or no prenatal screenings further contribute to the challenges of managing congenital malformations. Prenatal screenings, such as ultrasound examinations and genetic testing, are essential for detecting potential anomalies and providing early interventions. However, the lack of access to these screenings or the delay in receiving them due to limited resources and healthcare infrastructure can result in missed opportunities for identifying and managing congenital malformations [38]. Insufficient healthcare infrastructure in developing countries, especially in low socioeconomic strata, hinders the provision of adequate healthcare and prenatal services. Limited availability of healthcare facilities, shortage of healthcare professionals, and inadequate equipment and resources pose significant barriers to delivering quality care. As a result, individuals and families facing financial constraints and living in these areas often struggle to access the necessary medical interventions and specialized care required for managing congenital malformations effectively [39].

Early detection and appropriate medical interventions are crucial for minimizing the impact of congenital malformations. Timely identification of congenital disabilities allows healthcare providers to develop appropriate management plans, counsel and support affected individuals and their families, and offer interventions such as surgeries, therapies, or assistive devices. Without adequate access to healthcare and prenatal services, individuals in low socioeconomic strata are disadvantaged when receiving these essential services, which can significantly affect their health outcomes and quality of life [40]. Efforts to improve access to healthcare and prenatal services in developing countries should strengthen healthcare infrastructure, increase the availability of trained healthcare professionals, promote community-based healthcare models, and address financial barriers. Enhancing awareness about the importance of prenatal care and early detection of congenital malformations is also crucial. Individuals in low socioeconomic strata can receive the necessary support and interventions to manage congenital malformations effectively and improve their overall well-being by prioritizing access to healthcare services [41].

Environmental Factors and Their Impact on Congenital Malformations

Congenital malformations, or congenital disabilities, are structural or functional abnormalities during fetal development. While genetic factors play a significant role in congenital malformations, environmental factors can also profoundly impact them. Exposure to pollutants, toxins, and infectious agents can disrupt normal fetal development and increase the risk of congenital disabilities [42]. In the low socioeconomic strata of developing countries, individuals often face higher levels of environmental hazards. Air pollution, for example, is a significant concern in densely populated areas with limited ecological regulations and industrial emissions. Inhalation of particulate matter, heavy metals, and toxic gases can harm fetal development. These substances can cross the placental barrier and interfere with normal organ formation, leading to congenital malformations [43].

Industrial waste disposal is another environmental factor that poses risks to fetal development. Improper handling and disposal of industrial waste can contaminate soil, water bodies, and food sources. Pregnant women who consume contaminated food or water expose their developing fetus to harmful chemicals and toxins. These substances can disrupt cellular processes, interfere with organogenesis, and increase the likelihood of congenital disabilities [44]. Pesticides are commonly used in agriculture to control pests and increase crop yields. However, exposure to pesticide residues can be harmful, particularly during pregnancy. Pregnant women living in agricultural areas or working in farming occupations may face higher exposure to pesticides through inhalation, ingestion, or dermal contact. Some pesticides have been associated with an increased risk of congenital malformations such as neural tube defects and limb abnormalities [45].

Contaminated drinking water is a significant concern in many developing countries. Water sources contaminated with heavy metals, bacteria, viruses, or other pathogens can pose serious health risks to pregnant women and their developing fetuses. Ingesting contaminated water can lead to intrauterine infections, which are known to cause congenital malformations, including brain damage, hearing loss, and heart defects [46]. It is worth noting that the impact of environmental factors on congenital malformations is not limited to developing countries or low socioeconomic strata. Developed countries also face environmental challenges, and certain populations may be more vulnerable due to factors such as occupational exposure or living near industrial sites [47].

Nutritional Deficiencies and Their Association with Birth Defects

Nutritional deficiencies, such as inadequate intake of folic acid, iron, iodine, and other essential nutrients, are linked to an increased risk of congenital malformations. Low socioeconomic strata in developing countries often experience limited access to nutritious food, resulting in higher rates of nutritional deficiencies during pregnancy. Insufficient maternal nutrition can impair fetal development and contribute to congenital disabilities [48].

Understanding the socioeconomic determinants, access to healthcare, environmental factors, and nutritional deficiencies associated with congenital malformations in low socioeconomic strata is crucial for addressing and mitigating their impact. Efforts should focus on improving socioeconomic conditions, expanding healthcare access, reducing environmental risks, and promoting proper nutrition during pregnancy to reduce the occurrence and burden of congenital malformations in these populations [49].

Impact on individuals and communities

Physical and Psychological Consequences for Affected Individuals

Congenital malformations can have significant physical and psychological consequences for affected individuals. Physical disabilities, chronic health conditions, and functional limitations may impact their daily lives, mobility, and overall well-being. Moreover, individuals with congenital malformations may experience emotional and psychological challenges, including self-esteem issues, social isolation, and mental health concerns [50].

Economic Burdens and Challenges Faced by Families

Families of individuals with congenital malformations often face substantial economic burdens. Costs associated with medical care, surgeries, therapies, assistive devices, and ongoing treatments can financially strain families, particularly in low socioeconomic strata. These economic challenges may limit access to necessary healthcare services, educational opportunities, and other resources, exacerbating the impact on individuals and families [51].

Social Stigma and Discrimination Associated with Congenital Malformations

Individuals with congenital malformations may encounter social stigma and discrimination within their communities. Prejudice, stereotypes, and misconceptions about disability can lead to marginalization and exclusion. Limited social support, reduced educational and employment opportunities, and unequal access to social services can further perpetuate the cycle of stigma and discrimination [32].

Implications for Community Healthcare Systems and Resources

Congenital malformations in the low socioeconomic strata of developing countries place significant demands on community healthcare systems and resources. These systems often face challenges in providing adequate healthcare services, specialized care, rehabilitation, and long-term support for individuals with congenital malformations. Limited funding, infrastructure, and trained healthcare professionals can further strain the capacity to meet the needs of affected individuals and their families [16,20,21].

The impact of congenital malformations extends beyond the affected individuals to their families and communities. Understanding the physical, psychological, economic, and social implications is crucial for developing comprehensive support systems, inclusive policies, and community-based interventions that address the specific needs of individuals with congenital malformations. Efforts should promote inclusivity, reduce stigma and discrimination, and ensure equitable access to healthcare, education, and socioeconomic opportunities for affected individuals and their families [12,15,28].

Strategies and interventions

Overview of Existing Interventions and Initiatives

Various interventions and initiatives have been implemented to address congenital malformations in the low socioeconomic strata of developing countries. These include public health programs focusing on prenatal care, genetic counseling, vaccination campaigns, nutritional supplementation, and early detection through screening programs. Community-based initiatives, advocacy efforts, and awareness campaigns are crucial in raising awareness and promoting preventive measures [38,43,48].

Challenges and Limitations in Implementing Effective Strategies

Implementing effective strategies to address congenital malformations in low socioeconomic strata faces several challenges. Limited healthcare infrastructure, inadequate funding, workforce shortages, and insufficient access to specialized care hinder the delivery of interventions. Cultural beliefs, social barriers, and low health literacy may impede the adoption of preventive measures and utilization of available services [19,31,47].

Innovative Approaches for Prevention, Detection, and Management

Innovative approaches are being explored to enhance the prevention, detection, and management of congenital malformations. These include using technology for telemedicine, mobile health applications, and remote consultations to reach underserved populations. Community health workers, trained in identifying risk factors and providing basic interventions, can be crucial in delivering healthcare services. Additionally, advancements in genetic testing, prenatal screening, and precision medicine offer promising avenues for early detection and personalized management [52].

Importance of Multidisciplinary Collaboration and Partnerships

Addressing congenital malformations in low socioeconomic strata requires multidisciplinary collaboration and partnerships. Collaboration between healthcare professionals, policymakers, community organizations, non-governmental organizations (NGOs), and international agencies is essential to develop comprehensive strategies, share best practices, and leverage resources. Collaborative efforts can facilitate capacity building, knowledge transfer, and the implementation of sustainable interventions that address the complex challenges associated with congenital malformations [53].

By leveraging existing interventions, addressing challenges, embracing innovative approaches, and fostering collaboration, effective strategies can be developed and implemented to reduce the burden of congenital malformations in the low socioeconomic strata of developing countries. These efforts should prioritize community engagement, cultural sensitivity, and long-term sustainability to ensure lasting impact and improved outcomes for affected individuals and their families [54].

Ethical considerations and policy implications

Ethical Dilemmas in the Context of Congenital Malformations

The presence of congenital malformations in low socioeconomic strata raises various ethical dilemmas. These include issues related to resource allocation, prioritization of interventions, informed consent for medical procedures, genetic testing and counseling, and decision-making regarding the termination of pregnancies. Balancing individual autonomy, cultural beliefs, and societal well-being while ensuring equitable access to healthcare services poses ethical challenges that require careful consideration [55].

Importance of Equity and Access to Healthcare Services

Achieving equity and ensuring access to healthcare services for individuals with congenital malformations is crucial. Policies and interventions should prioritize reducing disparities and addressing barriers that hinder access to quality healthcare. This includes improving healthcare infrastructure, increasing the availability of specialized care and rehabilitation services, and addressing socioeconomic determinants contributing to unequal healthcare access [56].

Policy Recommendations for Improving Prevention and Care

Policy recommendations can significantly impact the prevention and care of congenital malformations in low socioeconomic strata. These may include strengthening healthcare systems, promoting early and comprehensive prenatal care, implementing screening programs, enhancing genetic counseling services, ensuring essential medications and nutritional supplements are available, and integrating disability-inclusive policies in education and employment. Policies should also address social determinants of health and support community-based initiatives that empower individuals and families [57].

Advocacy for Increased Awareness and Support

Advocacy is vital in raising awareness, reducing stigma, and promoting support for individuals with congenital malformations. Advocacy efforts should increase public awareness about the causes, prevention, and management of congenital malformations. They should also emphasize the importance of inclusive and supportive environments that empower individuals with congenital malformations to lead fulfilling lives. Advocacy can mobilize resources, engage stakeholders, and drive policy changes to improve the lives of affected individuals and their families [58].

Ethical considerations and policy implications should guide decision-making processes, promote equity, and address the unique challenges associated with congenital malformations in low socioeconomic strata. By prioritizing ethical principles, advocating for equitable access to healthcare, implementing evidence-based policies, and raising awareness, societies can reduce the burden of congenital malformations and promote the well-being and rights of affected individuals [59].

## Conclusions

Despite significant progress, several gaps in knowledge regarding congenital malformations in low socioeconomic strata remain. Further research is needed to understand better the specific risk factors and mechanisms underlying the higher prevalence rates observed in these settings. Additionally, more studies are required to assess congenital malformations' long-term outcomes, social determinants, and economic impact. Research focusing on the effectiveness and cost-effectiveness of interventions and the experiences and perspectives of affected individuals and their families would also contribute to the existing knowledge base. Congenital malformations in the low socioeconomic strata of developing countries represent a significant public health concern with far-reaching implications. It is imperative to address these challenges through a multi-faceted approach that encompasses preventive measures, early detection, accessible healthcare services, social support, and inclusive policies. By prioritizing equity, promoting awareness, and fostering collaboration among stakeholders, we can reduce the burden of congenital malformations, improve the quality of life for affected individuals, and create more inclusive and supportive communities.
